# Patient-Reported Outcomes and Psychosocial Impact of Vascular Malformations in Asian Patients

**DOI:** 10.3390/jcm14113799

**Published:** 2025-05-29

**Authors:** Hechen Jia, Hongyuan Liu, Xi Yang, Zi’an Xu, Lan Luo, Yuyan Zhang, Chen Hua, Xiaoxi Lin

**Affiliations:** 1 Department of Plastic & Reconstructive Surgery, Shanghai Ninth People’s Hospital, Shanghai Jiao Tong University School of Medicine, Shanghai 200011, China; hchnjia7130@alumni.sjtu.edu.cn (H.J.); liuhongyuan1994@sjtu.edu.cn (H.L.);; 2 Department of Hemangioma and Vascular Malformation, Plastic Surgery Hospital, Chinese Academy of Medical Sciences and Peking Union Medical College, Beijing 100144, China

**Keywords:** vascular malformations, psychosocial impact, patient-reported outcomes, OVAMA, PROMIS

## Abstract

**Background**: Vascular malformations (VAMs) impose multifaceted burdens extending beyond physical impairments to psychosocial dysfunction. While prior studies predominantly utilized generic quality-of-life instruments, disease-specific tools are critical for addressing heterogeneous symptom profiles and sociocultural variability, particularly in understudied Asian populations. This study investigated psychosocial impacts across pediatric and adult VAM patients via validated, condition-specific measures. **Methods**: A prospective cohort of 233 hospitalized VAM patients (114 pediatric patients, 119 adult patients) completed the OVAMA questionnaire, and 114 adult, 68 pediatric patients, and 115 parent-proxies completed corresponding PROMIS questionnaires. The subtypes included arteriovenous malformations (AVMs), venous/lymphatic/lymphovenous malformations (VMs/LMs/LVMs), port-wine stains (PWSs), and other vascular malformations. Statistical analyses (Mann–Whitney U test, Kruskal–Wallis test, linear regression) were used to evaluate associations between demographics, clinical characteristics, and psychosocial outcomes. **Results**: Compared with children, adults reported significantly greater distress related to general (*p* = 0.004) and appearance (*p* = 0.003) problems. Compared with AVM (*p* = 0.01) and PWS (*p* = 0.041) patients, VM/LM/LVM patients presented elevated general problem scores. Pain and bleeding were related to general problems, whereas temporary enlargement was related togeneral and appearance problems. The PROMIS results revealed that 42.1% of adults had below-normal psychosocial-positive scores, whereas 33% demonstrated abnormal psychosocial-negative scores. Pediatric self-reports were associated with higher anxiety and depression rates than parent proxies were, with the VM/LM/LVM subgroups reporting poorer family relationships (*p* = 0.0062) and life purposes (*p* = 0.0075). Treatment frequency was correlated with increased psychological stress in children (*p* = 0.007). **Conclusion**: VAMs significantly impair psychosocial functioning across all ages, with adults experiencing heightened distress and social role deficits. Pediatric patients with low-flow malformations (VMs/LMs/LVMs) face compound depressive symptoms and familial strain. Disease-specific tools such as OVAMA and PROMIS are essential for comprehensive assessments, guiding tailored interventions to address both physical and psychosocial burdens.

## 1. Introduction

Vascular malformations (VAMs) are predominantly congenital vascular anomalies originating from dysregulated embryologic angiogenesis. These lesions can lead to disfigurement, physical disabilities, and functional impairments. Disease progression may not only cause severe complications—including refractory ulceration, consumptive coagulopathy, life-threatening hemorrhage, and hemodynamic decompensation—but also impose profound psychosocial burdens, which disproportionately affect patients’ quality of life relative to clinical severity.

Previous studies have reported the psychological impacts of common infantile hemangiomas on affected children and their parents. Most findings indicate significant psychological burdens on caregivers, with minimal direct effects on children younger than 4–5 years. Timely intervention may prevent persistent psychological sequelae later in childhood [1]. For combined intracranial–extracranial vascular anomalies such as Sturge–Weber syndrome, extensive evidence has revealed neurocognitive and psychiatric abnormalities, including mood disorders, disruptive behavioral disorders, adjustment disorders, and substance abuse [2,3,4,5]. Although increasing attention has been given to the psychosocial and quality-of-life (QoL) impacts of vascular malformations, most studies rely on generic QoL instruments and lack validated disease-specific assessment tools [6,7,8,9]. Recently, the OVAMA international consensus group pioneered a patient-reported outcome measure for peripheral vascular anomalies, recommending the implementation of the Patient-Reported Outcomes Measurement Information System (PROMIS) for psychosocial and QoL evaluation [10,11,12,13,14].

Given the substantial sociocultural differences between Western and Asian populations, systematic investigations of psychosocial and QoL impacts in Asian patients with VAMs hold critical value for clinical staging, treatment indications, and outcome assessment. No prior studies have specifically addressed this demographic. The relationships among age, sex, classification, location, treatment history, and the psychosocial impact of VAMs remain unexplored. This study aims to analyze the psychosocial impacts of vascular malformations across pediatric and adult populations.

## 2. Methods and Materials

This study was approved by the Institutional Review Board, and written informed consent was obtained from all participants. This study was conducted in accordance with the Declaration of Helsinki (World Medical Association, 2013). We prospectively administered the OVAMA instrument and items selected from the Patient-Reported Outcomes Measurement Information System (PROMIS) to investigate adult and pediatric patients with VAMs hospitalized at our institution between August 2021 and September 2022.

### 2.1. The OVAMA Questionnaire

The OVAMA questionnaire is a disease-specific patient-reported outcome (PRO) instrument for vascular malformation patients. Developed through international consensus-building involving patients, clinicians, and methodological experts, this tool evaluates the core outcome set (COS), a standardized collection of outcome domains recommended for assessing therapeutic efficacy in specific populations, for vascular malformations [11,12]. OVAMA was specifically designed to measure disease-critical domains, with a principal focus on symptomatology and aesthetic manifestations of vascular anomalies. In this study, we implemented the OVAMA instrument to assess patient-reported disease impact and perform aesthetic evaluations among hospitalized vascular malformation patients at our institution. The instrument comprises four composite domains, i.e., general problems, general symptoms, head and neck symptoms, and appearance. Both general problems and appearance employ 5-point Likert scales. The former contains 2 items assessing overall distress (frequency and intensity), whereas the latter includes 9 items evaluating the cosmetic impact of vascular malformations (VAMs) on patients, i.e., size, swelling, color, texture, facial features, body features, staring, self-confidence, and satisfaction. Each score of the scale can be converted into a composite score via the following formula:

General problems: ((item 1 of the general symptoms scale + item 2 of the general symptoms scale)/2) × 20

Appearance: ((sum of all 9 items of the appearance scale)/9) × 20

In addition, the questionnaire uses general symptoms to survey pain, bleeding, fluid leakage, and temporary enlargement of the vascular malformation, whereas head and neck symptoms assess breathing, eyesight, hearing, swallowing, speech, chewing, taste problems, and saliva leakage.

### 2.2. Patient-Reported Outcomes Measurement Information System (PROMIS)

This study utilized the Patient-Reported Outcomes Measurement Information System (PROMIS) to assess VAM patients’ QoL. The PROMIS encompasses over 300 validated measurement tools for evaluating physical, mental, and social health in both the general population and adults and pediatric patients. Although another disease-specific validated questionnaire—PROVAM—also measures the psychosocial status of VAM patients, PROMIS contains more psychosocial domains and sets questions in more dimensions. For comprehensive evaluation of QoL in VAM patients, OVAMA and PROMIS were employed for general, symptomatic, appearance, and psychosocial assessments rather than PROVAM. Through a multidisciplinary consensus process involving VAM patients and clinicians specializing in vascular anomalies, we collaboratively selected PROMIS measures that could most likely be used to conduct a comprehensive evaluation of the disease-specific core outcome domains critical to VAM populations. The included item banks were as follows:

Adult item banks included psychosocial illness impact-positive, psychosocial illness impact-negative, managing social interactions, and ability to participate in social roles and activities.

The parent-proxy (ages 5–17) and pediatric (ages 8–17) item banks included positive affect, anxiety, depressive symptoms, psychological stress experiences, life satisfaction, mean and purpose, peer relationships, and family relationships.

The early childhood parent-report (ages 1–5) item banks included anxiety, anger/irritability, and depressive symptoms.

All PROMIS item raw scores were converted to T-scores using the Health Measures Scoring Service “https://www.assessmentcenter.net/ac_scoringservice (accessed on 26 June 2022)” by calibrating to the means of corresponding reference populations, which can be general or disease-specific, varying across items, and then categorized according to established PROMIS cutoff criteria. The reference population and score cut points of each item can be found online “https://www.healthmeasures.net/score-and-interpret/interpret-scores/promis (accessed on 30 June 2022)”.

### 2.3. Data Collection

All of the OVAMA and PROMIS questionnaires were administered electronically through QR code-accessible digital forms. The enrolled patients completed the surveys via mobile devices, with mandatory field validation implemented to prevent missing data from manual entry errors. The backend system enables the automated export of structured datasets in spreadsheet format for subsequent analysis.

### 2.4. Statistical Analysis

Categorical variables associated with baseline characteristics are described as frequencies and percentages. Nonparametric continuous variables are presented as medians with interquartile ranges (IQRs). For between-group comparisons, nonparametric tests were used: the Mann–Whitney U test for two independent samples and the Kruskal–Wallis test for multiple groups. Multivariate analysis was conducted through linear regression modeling. All statistical procedures were performed via SPSS Statistics version 30.0 (IBM Corp., Armonk, NY, USA), with a *p* < 0.05 threshold for statistical significance.

## 3. Results

### 3.1. OVAMA Questionnaire

A total of 233 patients and parent-proxies completed the OVAMA assessment, comprising 105 males and 128 females, with 114 pediatric and 119 adult participants. The cohort included the following vascular malformation subtypes: 119 arteriovenous malformations (AVMs), 58 venous/lymphatic/lymphovenous malformations (VMs/LMs/LVMs), 47 port-wine stains (PWSs), and 13 cases of combined VAMs or associated syndromes. The latter group included capillary malformation–arteriovenous malformation, capillary-lymphatic malformation, venous and arteriovenous malformation, Klippel–Trenaunay syndrome, and venous malformation (see Table 1).

**Figure 1:**
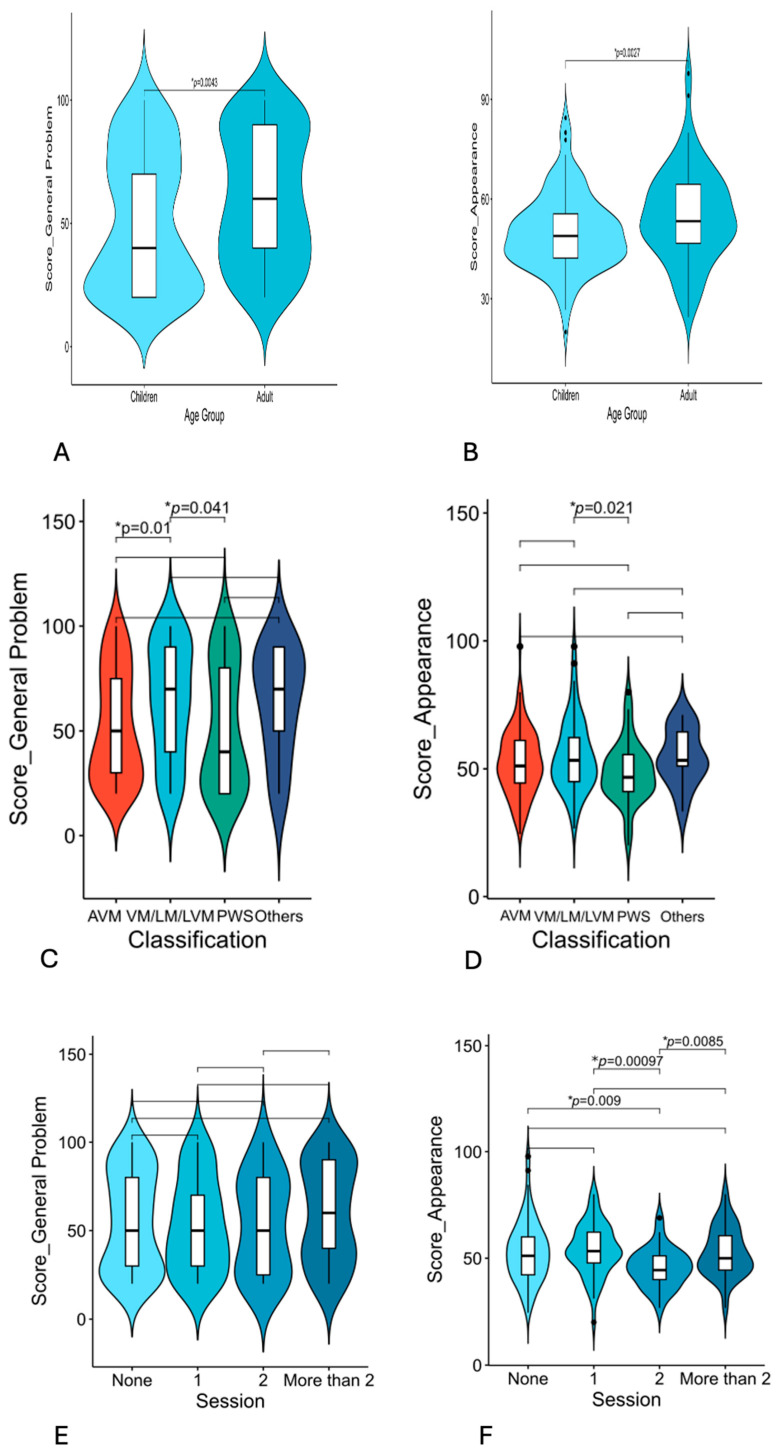
OVAMA general problem and appearance score of vascular malformation patients. (**A**,**B**) general problem and appearance score by age groups. (**C**,**D**) general problem and appearance score by classifications. (**E**,**F**) general problem and appearance score by treatment sessions. * *p* < 0.05. AVM = arteriovenous malformation, VM = venous malformation, LM = lymphatic malformation, LVM = lymphatic venous malformation, PWS = port-wine stain.

The median composite scores were 50 for general problems and 51 for appearance. Subgroup analysis revealed no significant sex-based differences in general or appearance-related concerns among vascular malformation patients. However, age-stratified comparisons demonstrated that adults were significantly more bothered by VAMs than were children with respect to both general problems (*p* = 0.004) and appearance-related issues (*p* = 0.003) (Table 1, Figure 1A,B). When categorized by malformation type, patients with venous, lymphatic, or lymphovenous malformations presented significantly elevated general problem scores compared with those with arteriovenous malformations (*p* = 0.01) and port-wine stains (*p* = 0.041) and higher appearance scores than did PWS patients (*p* = 0.021) (Figure 1C,D). Compared with patients in other anatomical subgroups, patients with lower limb or torso lesions presented numerically higher general problem scores; however, the difference was not statistically significant. No correlation was observed between the number of prior treatments and general distress scores. Notably, patients who had undergone two previous treatments reported significantly less appearance-related distress than did untreated patients (*p* = 0.009), those with one treatment (*p* < 0.001), and those with more than two treatments (*p* = 0.008) (see Table 1, Figure 1E,F).

Analysis of general symptoms revealed that in VAM patients, general problem scores were significantly associated with pain (*p* = 0.002), bleeding (*p* < 0.001), and temporary enlargement (*p* = 0.004), whereas appearance problems were correlated primarily with bleeding (*p* = 0.001) and temporary enlargement (*p* < 0.001) (see Table 2).

Further evaluation of head and neck symptoms revealed that patients with visual impairment and mastication difficulties presented elevated general problem scores (*p* = 0.01 and *p* = 0.03, respectively). Notably, specific appearance problems were strongly associated with visual impairment (*p* = 0.003) and sialorrhea (*p* = 0.007) (see Table 3).

Among 233 patients and parent-proxies who completed the OVAMA questionnaire, 167 (71.7%) also completed the PROMIS questionnaire.

### 3.2. Adult PROMIS Measurements

**Figure 2:**
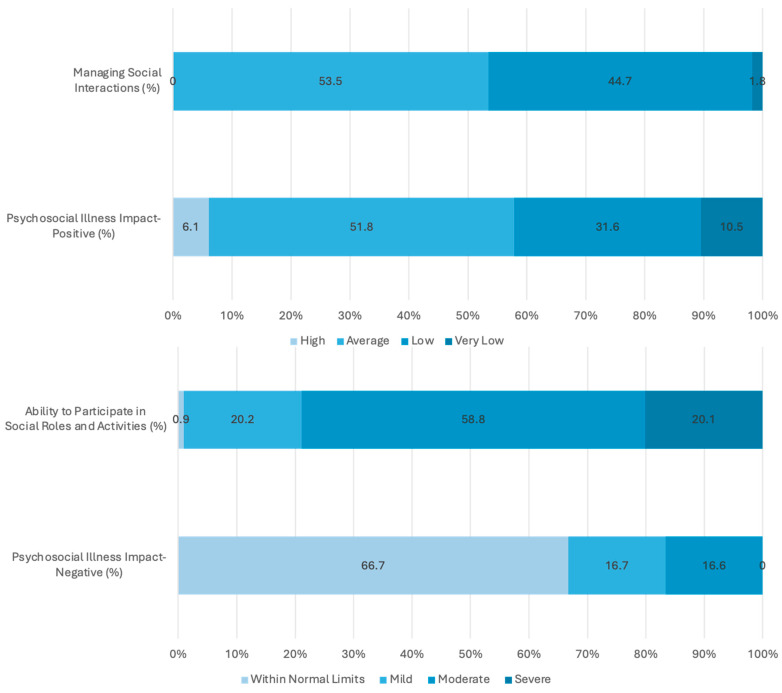
Adult PROMIS measures of patients with vascular malformations.

PROMIS measurements in 114 adult patients revealed that 42.1% of VAM patients had psychosocial illness, with positive T scores below the normative range of the reference population (<40). One-third of patients with mild or moderate psychosocial illness had negative T scores. The results indicated a marked reduction in positive psychological aspects and an increase in negative psychological burden. Approximately half of the patients scored below the normative range in managing social interaction. Nearly all patients demonstrated abnormalities in participating in social roles and activities, including approximately half of the patients with moderate abnormalities and 20.1% with severe abnormalities (Figure 2).

Subgroup analyses of VAM patients’ T scores across sex, classification, lesion location, and treatment history revealed that other vascular malformations and upper extremity vascular malformations had psychosocial illness impact scores below the average range. Venous, lymphatic, and lymphovenous malformations; PWS; other vascular malformations; and upper extremity vascular malformations scored below the average range in managing social interaction. No statistically significant differences were observed in the T scores of the four psychosocial items across the subgroups (Table S1).

### 3.3. Parent-Reported (Ages 5–17) and Pediatric (Ages 8–17) Measurements

**Figure 3:**
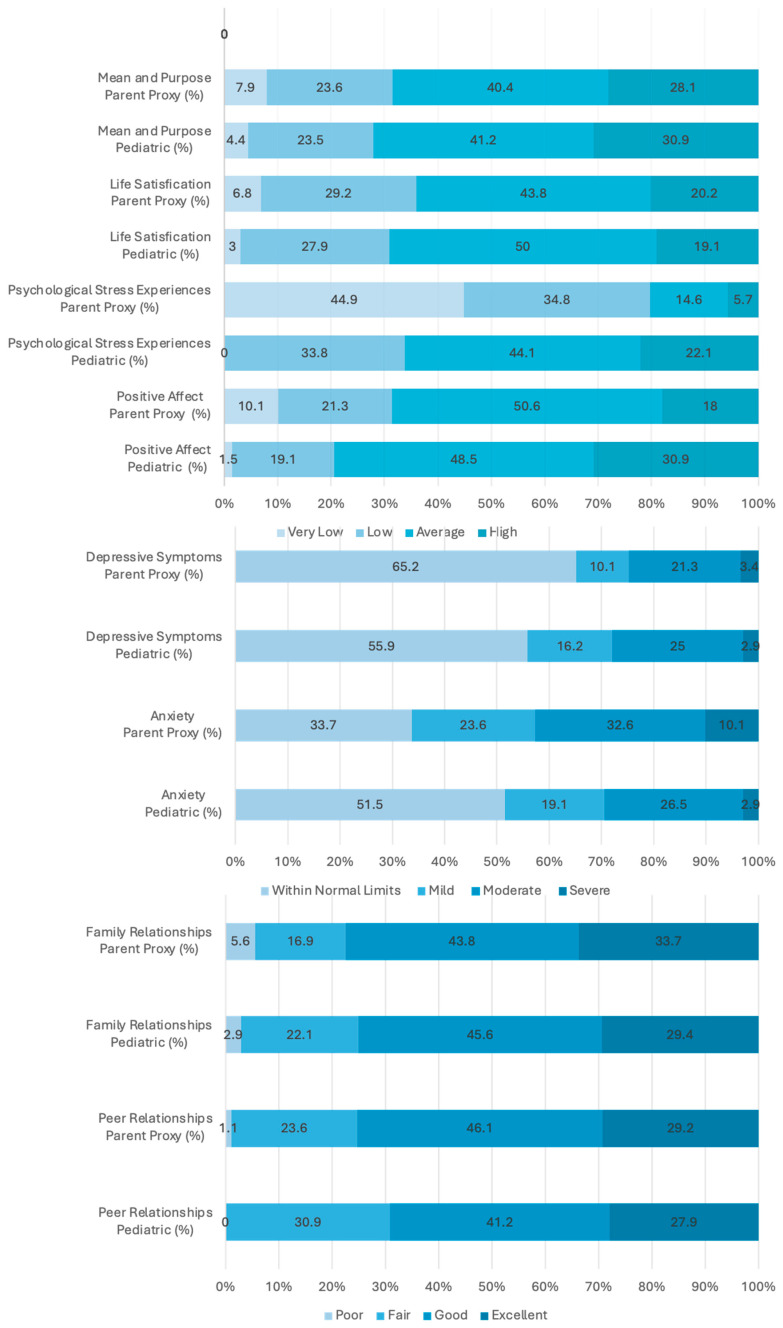
Parent-proxy PROMIS measures of pediatric patients with vascular malformations.

The evaluation results reported by the 89 parents and 68 children revealed that the parent-reported positive affect T score of approximately one-third of the children with VAM was lower than the normal range of the reference population, and approximately 20% of the children’s self-evaluation T scores were lower than those of the reference population, both indicating a significant decline in the children’s positive psychology; in terms of anxiety, approximately two-thirds of the parents’ evaluation results of their children showed abnormal anxiety, including 23.6% mild abnormalities, 32.6% moderate abnormalities, and 10.1% severe abnormalities. The proportion of anxious people in children’s self-evaluation results was greater than that in their parents’ evaluation. Approximately half of the patients had abnormal anxiety, 19.1% had mild abnormalities, 26.5% had moderate abnormalities, and 2.9% had severe abnormalities. Approximately one-third of parents’ evaluation results on the depression level of their children were abnormal, including 10.1% with mild abnormalities, 21.3% with moderate abnormalities, and 3.4% with severe abnormalities, while half of the children’s self-evaluations were abnormal, including 16.2% with mild abnormalities, 25% with moderate abnormalities, and 3.0% with severe abnormalities. In terms of psychological stress experience, approximately 20% of the T scores of both parental evaluations and children’s self-evaluations were higher than the average level, indicating that the disease can cause obvious psychological stress in children. In terms of social interaction, the evaluations of the children’s parents and the children themselves revealed that approximately 30% of the mean and purpose items had T scores below the average level, 30–40% of the life satisfaction T scores were below the average level, and poor peer and family relationships were rarely observed (Figure 3).

**Figure 4:**
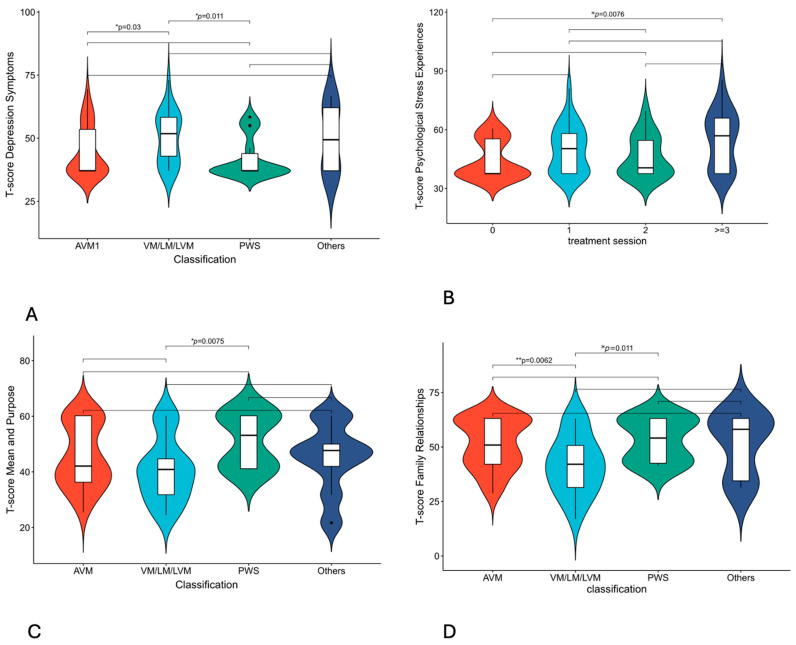
Parent-proxy PROMIS measures T-score of pediatric patients with vascular malformations. (**A**) T-score of depression symptoms by classifications. (**B**) T-score of psychological stress experience by treatment sessions. (**C**) T-score of mean and purpose by classifications. (**D**) T-score of family relationships by classifications. * *p* < 0.05; ** *p* < 0.01. AVM = arteriovenous malformation, VM = venous malformations, LM = lymphatic malformations, LVM = lymphatic venous malformations, PWS = port-wine stains.

According to the subgroup analysis results of parent-proxy evaluation, the depression T score of venous or lymphatic malformations was significantly greater than that of arteriovenous malformations and port-wine stains, and the difference was statistically significant (*p* = 0.03 and *p* = 0.011, respectively). In terms of psychological stress experience, the T score of children who had been treated more than three times was significantly greater than that of those who had not been treated (*p* = 0.007), suggesting that the increase in the number of treatments may be related to the increase in psychological stress in these children (Figure 4A,B). In terms of social aspects, the T scores of the mean and purpose items of patients with venous, lymphatic, and lymphovenous malformations were lower than those of patients with port-wine malformations (*p* = 0.0075), whereas the family relationships of children with venous, lymphatic, and lymphovenous malformations were significantly worse than those of patients with AVM or PWS (*p* = 0.0062, *p* = 0.011, respectively) (Figure 4C,D, Table S2). Subgroup analysis of the children’s self-assessment results revealed that children with venous and lymphatic malformations and port-wine stains were more likely to experience mild anxiety than other types were. The head and neck were mildly abnormal and significantly more common than other parts were. The anxiety levels of those who had not been treated and those who had been treated three times or more were all mildly abnormal. Depressive T scores in children with port-wine stains and untreated patients were mildly abnormal and higher than those in other disease types, but no statistically significant differences were found. No differences were found in the other measurements in the subgroup analysis (Table S3).

### 3.4. Parent-Reported Early Childhood (Age < 5) Measurement

The anxiety and depression results reported by 26 parents of younger children were abnormal in 30.8% of the children. Few children (7.7%) presented abnormalities related to anger irritability. Subgroup analysis did not reveal statistically significant differences between the groups (Table S4).

## 4. Discussion

This study utilized the OVAMA vascular malformation-specific scale and the PROMIS patient-reported outcome measurement system to identify significant psychological and social impacts of VMs on both adult and pediatric patients. Previous studies have employed generic quality-of-life instruments, such as the SF-36, Hospital Anxiety and Depression Scale (HADS), and visual pain scores, revealing reduced quality of life in vascular malformation patients compared with the general population [10,15,16]. Nevertheless, generic assessment tools are inadequate for accurately revealing the condition-specific psychosocial effects on individual patients because of the heterogeneity of the symptom spectrum and cosmetic impact of vascular malformation. This necessitates the development of disease-specific core domain sets and evaluation methodologies to assess the psychosocial burden comprehensively [11,12,17]. This study addresses prior limitations by employing the OVAMA scale and PROMIS measurement system to evaluate patient-reported general and appearance outcomes and multidimensional psychosocial effects in both adult and pediatric patients.

Analyses of the OVAMA scale’s general and appearance-related domains demonstrated that adult patients experienced significantly higher distress levels than pediatric patients did. PROMIS measurements indicated that while median T scores for psychological positive/negative impacts and social interactions in adults fell within normative ranges, at least one-third of patients presented abnormal levels. The majority of adults demonstrated a spectrum of mild to severe impairments in social role participation, regardless of disease type, lesion location, and treatment history, suggestive of universal psychological burdens and substantial social dysfunction across vascular malformations in adults. The combined effects of vascular anomalies, encompassing both disfiguring cosmetic manifestations and functional impairments in physical mobility, may substantially compromise psychosocial engagement. This underscores the clinical imperative for targeted interventions aimed at ameliorating both aesthetic and functional parameters to facilitate the restoration of normative social functioning.

Pain has consistently been a primary symptom of concern in low-flow vascular malformations, and many therapeutic interventions for these malformations aim to alleviate pain [18,19,20,21,22]. This study further revealed a significant correlation between pain and patients’ general distress, which is consistent with the conclusions of previous studies. England et al. identified correlations between venous malformations and pain and physical dysfunction via NRS pain scores and between PROMIS pain scores and physical items [7]. A meta-analysis by Nguyen et al. reported greater physical pain and psychological distress in vascular malformation patients than in the general U.S. population [6]. Stor et al. linked vascular malformation-related pain to compromised psychological health and quality of life via the OVAMA and PROMIS [23]. England et al. further demonstrated improved emotional and social well-being posttreatment using a vascular malformation-specific measurement tool [24]. Previous studies have focused predominantly on the pain associated with vascular malformation, whereas the psychosocial impacts of other symptoms remain understudied.

This study revealed that, in addition to pain, bleeding and transient lesion enlargement—critical markers of disease progression—also significantly contribute to both general and appearance-related concerns in VAM patients, indicating the need for specific evaluation and tailored clinical management of these symptoms to alleviate physical and psychological burdens and achieve better patient satisfaction [19,25]. For head and neck VAMs, the progression and treatment of orbital vascular malformations are associated with a high risk of blindness, and visual impairment has long been a critical concern for both patients and clinicians [26,27]. This study further confirms that visual impairment can simultaneously lead to both general and appearance-related distress in patients. Comprehensive ophthalmic evaluations, early intervention, and precise lesion-targeting therapy may help mitigate visual risk while significantly alleviating patients’ anxiety [28,29].

This study’s parent-proxy and pediatric reports revealed concordant psychological stress exposure levels but higher anxiety and depression rates in child self-evaluations, suggesting parental underestimation of the psychosocial impacts on their children. Subgroup analyses revealed greater depression levels in patients with low-flow (venous, lymphatic, or lymphovenous) malformations than in those with AVMs and port-wine stains and in patients with >3 treatments. Harvey et al. documented prevalent anxiety and depression in Klippel–Trenaunay syndrome patients and analyzed potential stressors [8]. Pang et al. reported elevated anxiety and depression via the HADS and visual pain scale and revealed improvements in mental health and social functioning [15,30]. Venous or lymphatic malformations, which are distinct from other vascular malformations, frequently manifest not only with cosmetic concerns but also with clinically significant limb pain, developmental deformities, and mobility restrictions. These multidimensional physical manifestations may correlate with the marked anxiety–depression comorbidity profiles observed in pediatric patients. While most children showed normal peer relationships, the VM/LM/LVM subgroups presented poorer family relationships and life purpose scores. It is worth noting that nearly one-third of children under 5 years of age reported experiencing anxiety and depression. Prior studies emphasize early intervention to prevent negative self-perception, regardless of cosmetic severity, and advocate addressing caregiver psychological impacts [31].

## 5. Limitations

The sample size of the present study is limited. Larger cohorts of vascular malformation patients might reveal distinct psychosocial status variations across subgroups that were not significant in this study. The present study cohort consisted primarily of hospitalized patients undergoing active treatment, who are more likely to exhibit severe disease than outpatient patients are. Additionally, as a specialized vascular anomaly center receiving complex referral cases, the observed psychosocial burden may overrepresent severe clinical phenotypes, introducing potential selection bias. With respect to methodological limitations, the lack of pre-post therapeutic comparisons and control groups in this study precluded further study regarding the relationship between symptom severity and patients’ quality of life (QoL). Ghaffarpour et al. validated the long-term improvement in health-related quality of life (HRQoL) among children with lymphatic malformations following sclerotherapy [32]. The collection of longitudinal OVAMA and PROMIS measurements before and after treatment may further elucidate how therapeutic interventions and symptomatic changes modulate patients’ psychosocial impact. Recent studies demonstrated the responsiveness of the OVAMA and PROVAM questionnaires to symptom and appearance changes after treatment in patients’ VAMs and low-flow vascular malformations, respectively [33,34]. These findings establish these validated patient-reported outcome measures as potential standard methods for assessing therapeutic efficacy.

In contrast to the universal applicability of OVAMA, PROMIS items were tailored to the cognitive abilities and social developmental stages among different ages, which potentially limit comparability across age groups. Furthermore, PROMIS-calibrated T scores—derived from U.S. population norms—may inadequately reflect Asian sociocultural contexts because of the absence of region-specific reference data. Cross-cultural disparities in psychological constructs and health perceptions could bias outcome interpretations.

## 6. Conclusions

This study demonstrated that vascular malformations significantly impact self-perceived appearance, psychological well-being, and social functioning in both adults and children, extending beyond their established physical and functional impairments. Patients with vascular malformations commonly exhibit psychological and social functional abnormalities, including diminished positive psychological states, anxiety and depressive symptoms, and impaired social relationships. Notably, adult patients experience significantly greater general and appearance problems than pediatric patients do, with pronounced deficits in social role participation. Pediatric patients, particularly those with low-flow vascular malformations, such as venous or lymphatic malformations, may have more severe depressive symptoms, poorer life prospects, and compromised family relationships, even compared to those with AVMs. This disparity may be attributed to more common chronic functional impairments at an early stage—such as pain and limb dysfunction—in low-flow malformations, whereas AVM-related complications, though more severe, predominantly manifest during advanced disease progression. Furthermore, increased treatment frequency may exacerbate depressive manifestations in children.

Consequently, comprehensive psychosocial assessments are important for determining treatment indications and evaluating the quality of life of patients with VMs.

## Figures and Tables

**Table 1:** Characteristics and OVAMA composite score of patients with vascular malformations.

	*n*	General Problems *	Appearance *
	233	50	51
Gender			
Male	105	60 [30–80]	51 [44–62]
Female	128	50 [30–80]	51 [42–59]
*p*		0.576	0.233
Age			
Children (<18 yr)	119	40 [20–70]	49 [42–55]
Adult	114	60 [40–90]	53 [46–64]
*p*		**0.004**	**0.003**
Classification			
AVM	119	50 [30–80]	51 [44–62]
VM/LM/LVM	58	70 [40–90]	53 [44–63]
PWS	43	40 [20–80]	47 [40–55]
Others	13	70 [45–90]	53 [48–65]
*p*		**0.036**	0.099
Location			
Head and Neck	157	50 [30–75]	51 [42–60]
Upper Limb	28	50 [32–87]	51 [30–75]
Lower Limb	37	70 [40–90]	51 [44–60]
Torso	11	80 [60–90]	58 [49–62]
*p*		0.070	0.756
Treatment Session			
None	99	50 [30–80]	51 [42–60]
1	55	50 [30–70]	53 [47–62]
2	27	50 [20–80]	44 [38–51]
More than 2	52	60 [40–90]	50 [44–62]
*p*		0.564	**0.014**

* Data presented are the median [quartile]. The bolds stand for *p* < 0.05.

**Table 2:** Multivariable regression of general symptoms and OVAMA composite score of patients with vascular malformations (*n* = 233).

		General Problems		Appearance	
	*n* (%)	*p*	OR (95% CI)	*p*	OR (95% CI)
NGS *	141 (60.5)				
Pain	50 (21.5)	**<0.001**	19.61 (11.35~27.87)	0.505	−1.359 (−5.37~2.65)
Bleeding	23 (9.9)	**0.017**	13.63 (2.41~24.84)	**0.002**	8.565 (3.12~14.01)
Leakage of fluid	16 (6.9)	0.744	2.28 (−11.44~15.99)	0.055	6.527 (−0.13~13.19)
Temporary enlargement	52 (22.3)	**0.001**	13.06 (5.13~20.99)	**<0.001**	9.055 (5.21~12.90)

* Number of general symptoms. The bolds stand for *p* < 0.05.

**Table 3:** Multivariable analysis of head and neck symptoms and OVAMA composite score of patients with vascular malformations (*n* = 157).

		General Problems		Appearance	
	*n* (%)	*p*	OR (95% CI)	*p*	OR (95% CI)
NHNS *	99 (63)	-	-	-	-
Breathing	5 (3.2)	0.80	2.99 (−20.81~26.80)	0.689	2.38 (−9.32~14.08)
Eyesight	16 (10.2)	**0.01**	18.17 (4.18~32.15)	**0.003**	10.67 (3.80~17.55)
Hearing	2 (1.3)	0.55	11.13 (−26.08~48.35)	0.561	5.39 (−12.91~23.68)
Swallowing	8 (5.1)	0.28	−14.48 (−41.12~12.16)	0.721	2.37 (−10.73~15.46)
Speech	7 (4.5)	0.11	18.33 (−4.10~40.76)	0.074	10.03 (−0.99~21.06)
Chewing	6 (3.8)	**0.03**	31.12 (2.68~59.57)	0.821	−1.60 (−15.58~17.84)
Taste	1 (0.6)	0.86	5.13 (−52.95~63.20)	0.460	−10.71 (−39.25~17.84)
Saliva leakage	13 (8.3)	0.22	10.98 (−6.54~28.50)	**0.007**	11.90 (3.29~20.51)

* Number of head and neck symptoms. The bolds stand for *p* < 0.05.

## Data Availability

The dataset used and analyzed in this study is provided within the Supplementary Information Files.

## Supplementary Materials

The following supporting information can be downloaded at https://www.mdpi.com/article/10.3390/jcm14113799/s1, Table S1: Median T-score and subgroup analysis of calibrated PROMIS psychosocial scale for adult vascular malformation; Table S2: Parent-proxy items T-score (PROMIS) in children (5–17 years old) with vascular malformation; Table S3: Pediatric items T-score (PROMIS) in children with vascular malformation (8–17 years old); Table S4: Early Childhood (<5 years old) Parent Report items T-score (PROMIS) in children with vascular malformation.

## Abbreviations

VAMs	Vascular malformations
VMs/LMs/LVMs	Venous/lymphatic/lymphovenous malformations
AVMs	Arteriovenous malformations
PWSs	Port-wine stains
KTS	Klippel–Trenaunay syndrome
