# Supplementary material for: Patient-Reported Outcomes and Psychosocial Impact of Vascular Malformations in Asian Patients
Source: J Clin Med. 2025 May 29;14(11):3799. doi: 10.3390/jcm14113799 (PMC12155985; doi:10.3390/jcm14113799)
Supplement: Supplementary file 1 [file jcm-14-03799-s001.zip › jcm-3628814-supplementary.pdf]

Supplementary Materials

Table S1. Median T-score and subgroup analysis of calibrated PROMIS psychosocial scale for adult vascular malformation.

	N	Psychosocial Illness Impact-Positive (%)	Psychosocial Illness Impact-Negative (%)	Managing Social Interactions (%)	Ability to Participate in Social Roles and Activities (%)
N=114	114	42.60	51.90	41.00	36.60
Gender					
Male	51	40.20	52.00	40.10	36.90
Female	63	43.00	51.30	41.20	35.50
P		0.135	0.461	0.212	0.440
Classification					
AVM	91	42.80	51.90	41.50	36.40
VM/LM/LVM	13	43.30	55.10	39.90	35.60
PWS	6	40.20	49.00	37.20	36.60
Others	4	38.50	50.90	35.60	37.30
P		0.414	0.846	0.147	0.870
Location					
Head	80	43.20	51.60	41.30	34.80
Upper Limb	15	35.60	53.30	36.60	38.00
Lower Limb	12	42.80	51.90	42.60	36.20
Torso	7	40.90	51.90	40.90	36.90
P		0.113	0.680	0.230	0.056
Treatment Session					
None	45	42.80	52.60	40.10	36.30
1	31	41.30	52.60	39.90	36.90
2	18	43.20	48.20	42.00	33.80
More than 3	20	42.40	51.20	42.00	37.60
P		0.951	0.390	0.895	0.332

Table S2. Parent-proxy items T-score (PROMIS) in children (5-17 years old) with vascular malformation.

	N	Positive Affect (%)	Anxiety (%)	Depressive Symptoms (%)	Psychological Stress Experiences (%)	Life Satisfaction (%)	Mean and Purpose (%)	Peer Relationships (%)	Family Relationships (%)
	89	46.70	53.20	42.90	49.30	42.40	42.10	47.50	49.80
Gender									
Male	45	48.10	53.20	43.30	52.20	42.40	42.10	49.00	50.90
Female	44	46.35	53.20	40.00	37.60	42.40	44.00	45.05	45.00
P		0.663	0.824	0.770	0.062	0.410	0.547	0.262	0.405
Classification									
AVM	41	49.00	52.70	37.10	46.70	42.70	42.10	50.70	50.90
VM/LM/LVM	25	45.80	55.70	51.80	52.20	42.00	40.90	44.00	42.10
PWS	14	42.15	53.65	37.10	37.60	42.00	53.15	51.35	54.10
Others	9	48.10	54.10	49.40	50.60	55.10	47.70	52.00	58.10
P		0.495	0.303	0.035	0.226	0.409	0.049	0.184	0.019
Location									
Head	57	49.00	53.20	37.10	37.60	42.40	43.30	50.70	50.70
Upper Limb	11	40.20	56.50	49.40	50.60	42.70	43.60	47.50	44.20

Lower Limb	15	45.40	55.70	48.20	52.10	42.20	42.00	44.00	48.20
Torso	6	53.20	43.90	40.00	47.45	44.40	44.70	48.95	46.50
P		0.073	0.395	0.285	0.664	0.980	0.852	0.259	0.337
Treatment Session									
None	22	49.00	52.95	37.10	37.60	42.70	51.10	52.05	63.10
1	26	46.45	55.15	44.50	50.35	41.90	40.90	45.05	46.45
2	16	40.65	48.60	37.10	40.55	43.90	43.15	47.85	50.55
More than 3	25	49.00	54.10	43.30	56.90	42.00	42.00	47.50	49.50
P		0.645	0.510	0.364	0.038	0.336	0.078	0.468	0.146

Table S3. Pediatric items T-score (PROMIS) in children with vascular malformation (8-17 years old).

	N	Positive Affect (%)	Anxiety (%)	Depressive Symptoms (%)	Psychological Stress Experiences (%)	Life Satisfaction (%)	Mean and Purpose (%)	Peer Relationships (%)	Family Relationships (%)
	68	49.30	45.10	45.30	50.35	44.40	43.10	45.75	46.5
Gender									
Male	38	49.30	45.10	45.60	50.35	44.45	43.10	46.25	46.5
Female	30	48.90	47.90	44.75	49.45	44.40	43.45	45.75	46.5
P		0.631	0.796	0.695	0.985	0.233	0.758	0.807	0.292
Classification									
AVM	30	47.70	42.00	49.85	48.60	44.40	42.10	48.45	46.1
VM/LM/LVM	19	49.20	51.30	43.00	50.80	44.40	43.10	45.70	46.5
PWS	12	48.25	56.35	54.30	59.25	44.40	43.25	41.90	46.1
Others	7	60.70	43.10	35.20	46.50	50.40	60.60	64.40	58.7
P		0.394	0.282	0.204	0.322	0.369	0.474	0.089	0.251
Location									
Head	44	49.25	52.10	47.15	50.35	44.40	43.55	48.80	46.45
Upper Limb	8	48.60	38.30	35.20	45.15	44.40	43.80	45.70	46.05
Lower Limb	12	50.55	37.40	39.85	44.95	44.40	43.65	45.70	46.5
Torso	4	50.30	39.30	42.35	55.60	44.45	42.00	57.45	52.8
P		1.000	0.246	0.865	0.894	0.882	0.917	0.702	0.904
Treatment Session									
None	17	49.30	52.10	53.30	46.80	44.40	43.10	45.70	46.5
1	18	48.20	44.10	42.95	54.05	44.35	43.10	45.70	45.5
2	14	49.55	33.50	35.20	39.90	45.75	43.25	48.00	55.2
More than 3	19	49.80	50.60	46.10	52.10	44.40	44.90	48.10	46.5
P		0.971	0.878	0.910	0.879	0.666	0.445	0.865	0.834

Table S4. Early Childhood (< 5 years old) Parent Report items T-score (PROMIS) in children with vascular malformation.

	N	Parent Report Anxiety (%)	Parent Report Anger/Irritability (%)	Parent Report Depressive Symptoms (%)
	26	57.35	56.15	49.60
Gender				
Male	14	58.80	57.05	51.80
Female	12	54.25	55.75	48.45
P		0.226	0.503	0.189
Classification				
AVM	3	58.90	59.40	48.70
VM/LM/LVM	5	56.70	52.80	52.90
PWS	16	54.80	55.75	49.20
Others	2	56.30	65.25	44.55
P		0.626	0.512	0.893
Location				
Head	18	58.35	56.50	49.90
Upper Limb	2	55.15	54.10	45.70
Lower Limb	5	58.90	56.50	49.70

Torso	1	52.50	56.20	48.20
P		0.752	0.864	0.926
Treatment Session				
None	9	56.70	57.60	48.70
1	8	51.80	55.65	51.80
2	4	61.10	55.75	47.25
More than 3	5	58.90	59.30	49.50
P		0.615	0.877	0.804
